# Brain Atrophy and Reorganization of Structural Network in Parkinson's Disease With Hemiparkinsonism

**DOI:** 10.3389/fnhum.2018.00117

**Published:** 2018-03-27

**Authors:** Xiaojun Xu, Xiaojun Guan, Tao Guo, Qiaoling Zeng, Rong Ye, Jiaqiu Wang, Jianguo Zhong, Min Xuan, Quanquan Gu, Peiyu Huang, Jiali Pu, Baorong Zhang, Minming Zhang

**Affiliations:** ^1^Department of Radiology, Second Affiliated Hospital of Zhejiang University School of Medicine, Hangzhou, China; ^2^Department of Neurology, Second Affiliated Hospital of Zhejiang University School of Medicine, Hangzhou, China; ^3^Department of Radiology, Zhejiang Provincial People's Hospital, People's Hospital of Hangzhou Medical College, Hangzhou, China

**Keywords:** Parkinson's disease, hemiparkinsonism, deformation-based morphometry, structural correlation network, magnetic resonance imaging

## Abstract

Hemiparkinsonism duration in patients with Parkinson's disease (PD) is a key time window to study early pathology of PD. We aimed to comprehensively explore the alterations of deformation and structural network in PD patients with hemiparkinsonism, which could potentially disclose the early biomarker for PD. Thirty-one PD patients with hemiparkinsonism and 37 age- and gender- matched normal controls were included in the present study. First of all, we normalized the left hemisphere of structural images as the contralateral side to the affected limbs. Deformation-based morphometry (DBM) was conducted to evaluate the brain atrophy and/or enlargement. structural networks were constructed by thresholding gray matter volume correlation matrices of 116 regions and analyzed using graph theoretical approaches (e.g., small-worldness, global, and nodal measures). Significantly decreased deformation values were observed in the temporoparietal regions like bilateral middle temporal gyri, ipsilateral precuneus and contralateral Rolandic operculum extending to supramarginal and postcentral gyri. Lower deformation values in contralateral middle temporal gyrus were negatively correlated with higher motor impairment which was dominated by akinesia/rigidity. Moreover, nodal reorganization of structural network mainly located in frontal, temporal, subcortex and cerebellum was bilaterally explored in PD patients with hemiparkinsonism. Increased nodal properties could be commonly observed in frontal lobes. Disruption of subcortex including basal ganglia and amygdala was detected by nodal local efficiency and nodal clustering coefficient. Twelve hubs, mainly from paralimbic-limbic and heteromodal networks, were disrupted and, alternatively, 14 hubs, most of which were located in frontal lobes, were additionally detected in PD patients with hemiparkinsonism. In conclusion, during hemiparkinsonism period, mild brain atrophy in the temporoparietal regions and widespread reorganization of structural network, e.g., enhanced frontal function and disruption of basal ganglia nodes, occurred in both hemispheres. With our data, we can also argue that MTG contralateral to the affected limbs (expressing clinically verified brain atrophy) might be a potential living biomarker to monitor disease progression. Therefore, the combination of DBM and structural network analyses can provide a comprehensive and sensitive evaluation for potential pathogenesis of early PD patients with hemiparkinsonism.

## Introduction

Parkinson's disease (PD) is becoming acknowledged as a multi-system disease gradually involving the whole brain following an ascending pattern from the lower brainstem to cortical areas (Braak et al., 2003; Braak and Del, 2008). At the clinical stage, loss of dopaminergic neurons within substantia nigral pars compacta is commonly believed to be a hallmark for PD with subsequently degenerative alterations in the neocortex (Fearnley and Lees, 1991; Braak and Del, 2008). On account of lacking definitive validated markers, clinical expert opinions have been the gold standard diagnostic technique (Postuma et al., 2015), which, to some extent, has led to a low accuracy of early disease diagnosis (Adler et al., 2014). Therefore, investigating early alterations of *in vivo* PD brains would contribute to understanding initial pathological phenotypes and capturing living biomarkers.

PD classically first manifests in one hemibody before affecting both sides (a condition known as hemiparkinsonism; Lees et al., 2009). Thus, the time period in which hemiparkinsonism takes place, with or without axial involvement, is a key time window for studying early pathology in PD (Djaldetti et al., 2006). High resolution MRI can accurately measure changes in brain structure. A growing body of neuroimaging evidence has established a notion that progressive development of parkinsonian brains across disease stages occurs in PD (Agosta et al., 2013; Luo et al., 2015). Though early PD patients including those who suffered from hemiparkinsonism were investigated (Borghammer et al., 2010; Agosta et al., 2013; Luo et al., 2015), studies have rarely tried to customize experimental protocols focusing on hemiparkinsonism except for a recent study showing bilaterally widespread functional alterations of the cortico-striatal-thalamic network (Agosta et al., 2014). While such evidence is far from explaining the disease substrates of PD hemiparkinsonism, the exploration of the structural basis for disease unilaterality in hemiparkinsonism might lead to an improved understanding of the underlying pathogenesis of early PD.

High resolution T1-weighted measurement allows for the interrogation *in vivo* of the exquisite structural alterations. Deformation-based morphometry (DBM) is a fully automatic intensity-based algorithm and can detect regional changes in the whole brain and requires no a priori knowledge of gray matter (GM) or white matter distributions throughout the brain (Ashburner et al., 1998; Gaser et al., 1999). The nonlinear image registration algorithm allows for a better detection of subtle GM differences in comparison with voxel-based morphometry (Ashburner and Friston, 2000). Atrophy in basal ganglia, fronto-temporal regions, occipital lobe and cerebellum had been detected by DBM in PD patients with different stages (Borghammer et al., 2010; Zeighami et al., 2015) and, recently, DBM also explained the progressive atrophy of PD brains along disease exacerbation (Fereshtehnejad et al., 2017). Moreover, structural correlation network (SCN) proposes another sensitive avenue of research to explore brain development and diseases (He et al., 2007, 2009; Zielinski et al., 2010; Pereira et al., 2015; Yadav et al., 2016). Reduced global efficiency and reorganization of network hub in SCN constructed by cortical thickness could be observed in PD patients with mild cognitive impairment (Pereira et al., 2015). As cortical volume is the product of cortical thickness and surface area, GM segmented by volume-based morphometry could be used to construct large-scale SCN which could reveal intrinsically structural organizational principles in the human brain (Hosseini and Kesler, 2013). We hoped that volume-based SCN could provide new information about the potential pathogenesis of PD patients with hemiparkinsonism other than DBM.

To date, no study has been performed to investigate the changes in deformation or intrinsic structural organization in hemiparkinsonism patients. We hypothesized that, by comprehensively exploring the underlying alterations of DBM and SCN properties in PD patients with hemiparkinsonism, we could disclose an early biomarker for PD.

## Materials and methods

All participants signed informed consent forms in accordance with the approval of the Medical Ethic Committee of the Second Affiliated Hospital of Zhejiang University School of Medicine.

One hundred and nineteen right-handed PD patients and 42 right-handed normal controls were initially recruited from the Second Affiliated Hospital of Zhejiang University School of Medicine. Diagnosis of PD was made by an experienced neurologist (B. Z.) according to UK Parkinson's Disease Society Brain Bank criteria (Hughes et al., 1992). Normal controls and PD patients with a history of other neurologic or psychiatric disorders, brain trauma, or general exclusion criteria for MR scanning were excluded from this study. Clinical assessments (Unified Parkinson's Disease Rating Scale (UPDRS), and the Mini-Mental State Examination (MMSE) scores, etc.) and image scanning were obtained from all participants. And, for PD patients taking anti-parkinsonian drugs, the above examinations were carried out after withholding all anti-parkinsonian medicine overnight (at least 12 h). In total, we screened 41 PD patients who were assigned to Hoehn-Yahr stage 1 or 1.5 with potentially unaffected limbs. After initial image quality and clinical data checking, we excluded an additional three PD patients with significant artifacts, five PD patients without unilateral preference of motor impairment, and one normal control with missing clinical data. Although all the patients recruited suffered from unilateral symptom, axial motor impairment could be generally observed. Therefore, we respectively recorded total motor impairment, motor impairment from affected limbs and axial motor impairment. For the heterogeneity of motor impairment, the UPDRS motor tremor score (sum of items 20 and 21) and the UPDRS motor akinesia/rigidity score (sum of items 22–27 and 31) were separately calculated according to the previous documents (Kang et al., 2005; Guan et al., 2017a,b). Thirty-three PD patients and 41 normal controls were taken into imaging analysis. According to MMSE estimated by the criteria suitable for Chinese population (Katzman et al., 1988; Zhang et al., 1990), all 74 subjects had a normal global cognition.

### MRI scanning

All 74 participants were scanned in a 3.0 Tesla MRI machine (GE Discovery 750) equipped with an eight-channel head coil. During MRI scanning, the head of each subject was stabilized with restraining foam pads. Earplugs were provided to reduce the noise during scanning. Structural T1 images were acquired using a Fast Spoiled Gradient Recalled sequence: repetition time = 7.336 ms; echo time = 3.036 ms; inversion time = 450 ms; flip angle = 11 degrees; field of view = 260 × 260 mm^2^; matrix = 256 × 256; slice thickness = 1.2 mm; 196 continuous sagittal slices.

### Imaging processing

Before imaging processing, brain images from 10 patients with left limbs affected were flipped from right to left, therefore, left hemisphere was defined as *contralateral* side to the affected limbs.

T1 structural images were first preprocessed by using Computational Anatomy Toolbox (CAT 12) (http://dbm.neuro.uni-jena.de/cat12/) within SPM 12. All these images were corrected for bias, noise and intensity, segmented into GM, white matter and cerebrospinal fluid, and registered to the Montreal Neurological Institute (MNI) standard space tissue probability maps. A partial volume estimation was extended to account for mixed voxels with two tissue types and spatial normalization using DARTEL was conducted. In addition, the Jacobian determinant for each voxel was computed in the normalized space, allowing for a direct estimation of the percentage change in volume in each voxel (DBM). Based on the data, total intracranial volume (TIV) was estimated. Because the inhomogeneity of MRI data is crucial for detecting real and subtle disease-related alterations, quantitative image quality was checked before further analysis using Mahalanobis distance, mean correlation and weighted overall image quality algorithms which were implemented into CAT 12. Raw images from a noticeable lower quality (below two standard deviations) were rechecked and finally excluded (two PD patients and four normal controls). Finally, the obtained preprocessed volume-based and DBM data from 31 PD patients (with a mean age of 57.1 ± 8.4 years) and 37 normal controls (with a mean age of 57.7 ± 7.9 years) were smoothed with an 8 mm full width at half maximum.

### Structural graph construction

**Figure 1:**
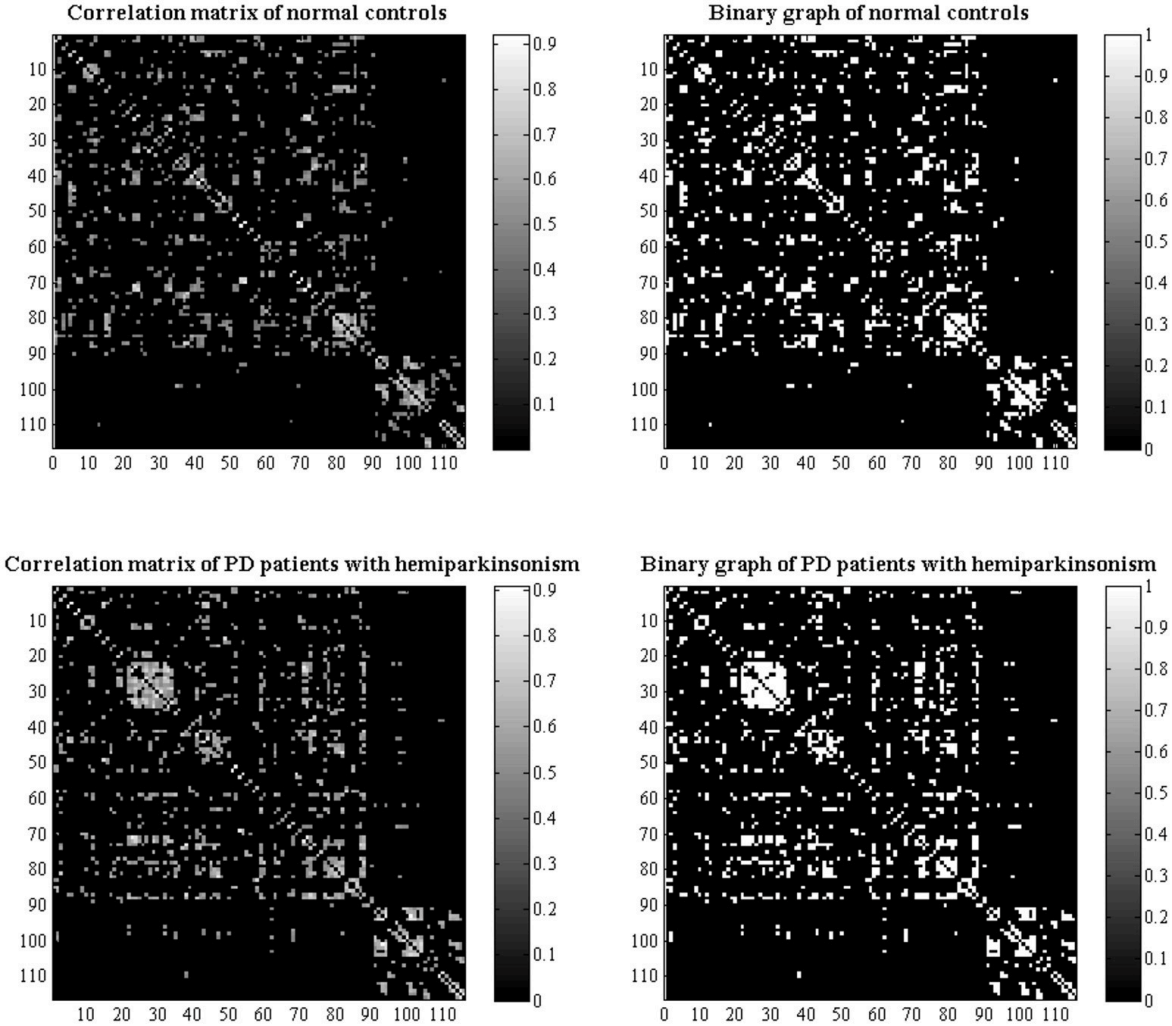
Structural correlation matrices with 116 × 116 in normal controls and PD patients with hemiparkinsonism were acquired. The left column showed the weighted correlation matrices while the right column showed the corresponding binary correlation matrices. Based on the weighted correlation matrices, we used a range of densities from 0.1 to 0.5 with an interval of 0.02 to construct network graphs respectively.

Modulated, normalized GM maps from the above voxel-based morphometry were implemented into graph analysis toolbox (GAT) (http://ncnl.stanford.edu/tools.html) (Hosseini et al., 2012). First, 116 ROIs including bilateral cortical, subcortical and cerebellar GM created using the Automatic Anatomic Labeling (AAL) atlas were used to extract the average volume within each ROI. Then, a structural matrix with 116 × 116 on group-level was constructed for each group (Figure 1) by using Pearson's correlation due to partial correlation is not suitable for studies with a sample size smaller than the number of ROIs (Zalesky et al., 2012). The effect of covariates of nuisance including age, sex and TIV was removed by performing a linear regression analysis. An edge between each pair of nodes was introduced when the correlation strength between the corresponding brain regions exceeded a certain threshold. Since some studies have shown that different numbers of edges in networks could interfere with detecting network organization (He et al., 2009; Rubinov and Sporns, 2010; Cao et al., 2014), we calculated the constructed correlation matrices at a range of network densities (from the estimated minimum density of 0.01 to maximum density of 0.05 with an interval of 0.02) for comparing the network topologies across that range. Here, a specific density means the fraction of active connections to all possible connections.

#### Small-world parameters

The small-worldness (Sigma) of a complex network has two key metrics: the clustering coefficient (C) and the characteristic path length (L). To evaluate the topology of the brain network, these parameters were compared to the corresponding mean values of a benchmark random graph (20 generated null networks). In a network with small-world, the clustering coefficient is significantly higher than that of random networks (Gamma = C/C_rand_ > 1) while the characteristic path length is comparable to random networks (Lambda = L/L_rand_ ≈ 1). The parameter of small-worldness Sigma equal to Gamma/Lambda would be greater than 1 (Watts and Strogatz, 1998; Liao et al., 2017).

#### Network measures

Here, we calculated commonly used global measures including mean local efficiency and global efficiency and regional measures including nodal betweenness centrality, nodal degree centrality, nodal clustering coefficient, nodal local efficiency and hub. Global efficiency indicates global efficiency of parallel information transfer in a network and mean local efficiency of the network measures how efficient communication is among the first neighbors of a given node when it is removed (Watts and Strogatz, 1998; Latora and Marchiori, 2001). Nodal degree centrality means information communication ability of a given node in the network; nodal betweenness centrality characterizes the effect of a given node on information flow between other nodes; nodal clustering coefficient of a given node measures the likelihood that its neighborhoods are connected to each other; nodal local efficiency for a given node measures how efficient the communication is among the first neighbors of this node when it is removed (Freeman, 1978; Rubinov and Sporns, 2010; Liao et al., 2017). Therefore, nodal degree centrality and betweenness centrality reflect how densely connected a given node is in the whole constructed network while nodal local efficiency and clustering coefficient are more likely to reflect nodal function within local subgraph. Finally, hubs are crucial components for efficient communication in a network. A node is considered as a hub if its nodal degree centrality is one SD higher than the mean network degrees. All these measures were originated from the codes developed in the Brain Connectivity Toolbox (Rubinov and Sporns, 2010).

### Statistical analysis

The normal distribution of data (e.g., age, education, MMSE and TIV) was confirmed using the one-sample Kolmogorov–Smirnov test. Differences in the age, sex, education, UPDRS III score, MMSE and TIV between groups were compared with unpaired *t*-test or Pearson chi-square appropriately. *P* < 0.05 was regarded as significant. These statistical analyses for clinical and demographic data were performed with statistical software (IBM SPSS Statistics v19.0; IBM Co., Armonk, NY, USA).

Voxel-wised statistical analysis of DBM data from two groups was conducted by using unpaired *t*-test, in which age, sex and MMSE score were regarded as covariates of no interest. For multiple comparisons correction, we detected the intergroup difference by using strict false discovery rate. However, no significant cluster was observed. Thus, to appropriately balance the type I and type II errors (Lieberman and Cunningham, 2009), an uncorrected *P* < 0.001 with cluster size > 200 was considered as an exploratory detection (Luo et al., 2015). Signals from the significant clusters between the comparisons of PD patients and normal controls were also extracted to test the correlations with clinical motor impairment scores including UPDRS III score, score from affected limbs, score of axial motor impairment, akinesia/rigidity and tremor scores.

In order to test the statistical significance of the between-group differences in network topology and regional network measures, a nonparametric permutation test with 1000 repetitions was used. Since comparing the networks at different densities results in multiple comparisons, a summary measure using area-under the curve (AUC) analysis was quantified. The curves extracted from thresholding across a range of densities were used. Each of these curves depicted the changes in a specific network measure (for each group) as a function of network density. As these correlative analyses of SCN were exploratory, we considered *P* < 0.05 as significant (Hosseini et al., 2012).

## Results

### Participants

No significant differences in age (*P* = 0.761), sex (*P* = 0.381), education (*P* = 0.432), and MMSE (*P* = 0.14) were found between PD patients and normal controls. The mean UPDRS III score in normal controls was 0.25 ± 0.6 while that in PD patients was 14.2 ± 5.7 (*P* < 0.001). The detailed subscales of UPDRS III score including akinesia/rigidity score, tremor score, motor score from the affected limbs and axial score were 8.7 ± 3.8, 2.3 ± 1.5, 8.8 ± 3.4, and 5.3 ± 2.8 respectively in PD patients. Due to patients with hemiparkinsonism being at a clinically early stage, no significant difference in TIV (*P* = 0.618) was observed. Detailed information could be seen in Table 1.

**Table 1:** Demographical and clinical information of participants.

	**Patients**	**Normal controls**	***p***
Number (male/female)	31 (15/16)	37 (14/23)	0.381
Right/left onset	21/10	–	–
Age, year	57.1 ± 8.4	57.7 ± 7.9	0.761
Education, year	10.23 ± 4.1	11.0 ± 3, 9	0.432
Disease duration, year	3.4 ± 3.8	–	–
UPDRS III score	14.2 ± 5.7	0.25 ± 0.6	<0.001
Akinesia/rigidity score	8.7 ± 3.8	–	–
Tremor score	2.3 ± 1.5	–	–
Motor score from affected side	8.8 ± 3.4	–	–
Axial score	5.3 ± 2.8	–	–
Hoehn-Yahr stage	1.2 ± 0.2	–	–
MMSE	28.1 ± 1.9	28.7 ± 1.6	0.140
Total Intracranial Volume, cm^3^	1481.7 ± 118.9	1496.4 ± 123.1	0.618

### Brain atrophy in PD patients with hemiparkinsonism

**Figure 2:**
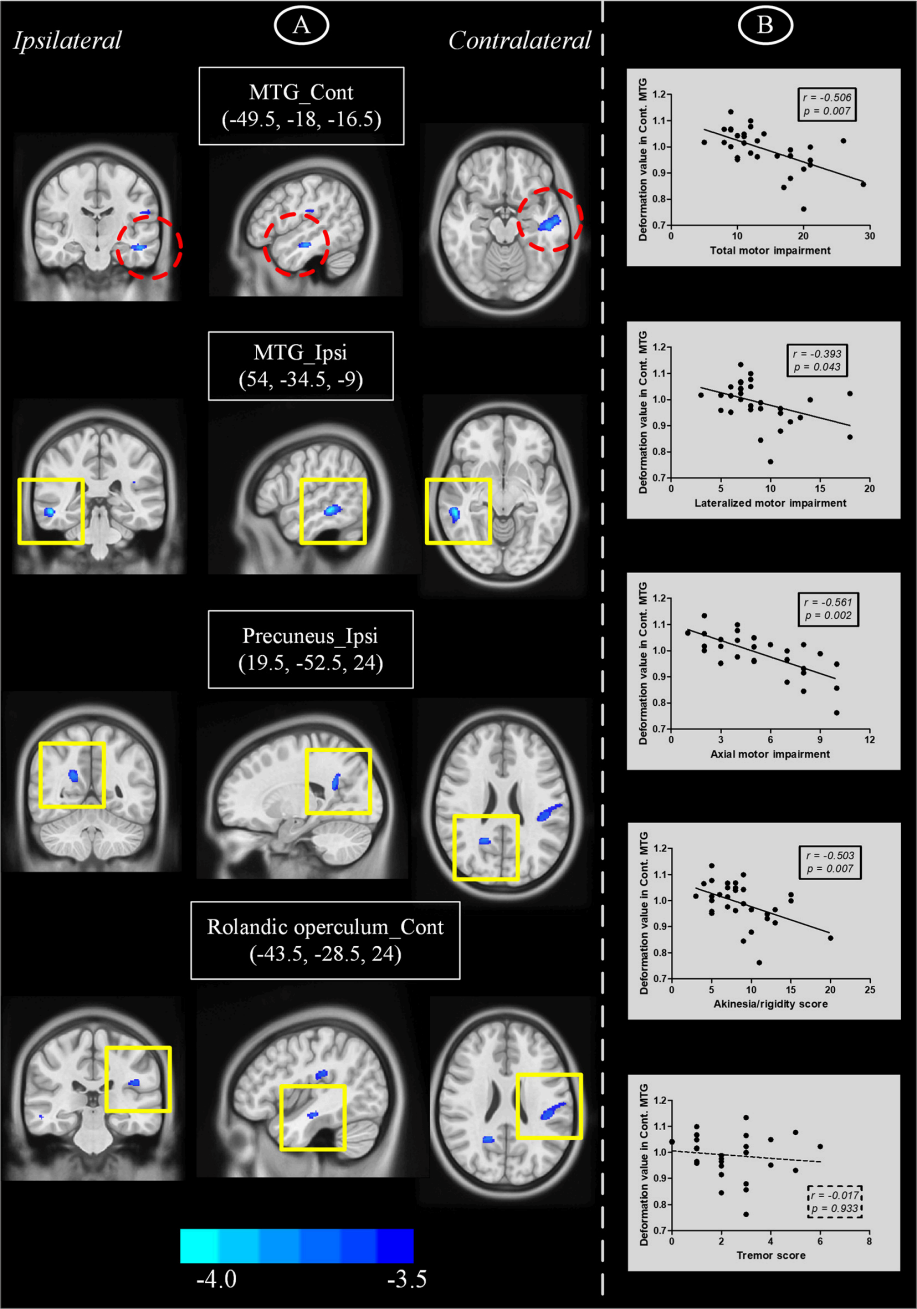
**(A)** Brain atrophy in PD patients with hemiparkinsonism compared with normal controls as measured by deformation-based morphometry. **(B)** Plots showing that decrease of deformation value in contralateral MTG was correlated with motor impairment. Cont, contralateral; ipsi, ipsilateral; MTG, middle temporal gyrus.

As only PD patients at an early clinical stage with unilateral symptoms were recruited, we detected mild atrophy in temporoparietal brain regions (Figure 2A and Table 2), e.g., bilateral middle temporal gyri (MTG) (ipsilateral side, peak MNI coordinate [54, −34.5, −9], *T* = −5.03, cluster size = 285; contralateral side, peak MNI coordinate [−49.5, −18, −16.5], *T* = −4.50, cluster size = 333), ipsilateral precuneus (peak MNI coordinate [21, −52.5, 25.5], *T* = −4.21, cluster size = 181) and Rolandic operculum (peak MNI coordinate [−43.5, −28.5, 24], *T* = −3.97, cluster size = 114) extending to supramarginal gyrus (cluster size = 50) and postcentral gyrus (cluster size = 42). No other brain atrophy or enlargement was observed.

**Table 2:** Regional brain atrophy in patients with Parkinson's disease compared with normal controls.

**Cluster number**	**Region**	**Ipsilateral/Contralateral**	**Cluster size**	**Peak MNI coordinate**			**Peak *T*-value**
				**X**	**Y**	**Z**	
Cluster 1	*Temporal lobe*	Contralateral	439	−49.5	−18	−16.5	−4.5
	Temporal middle gyrus	Contralateral	333				
Cluster 2	*Temporal lobe*	Ipsilateral	349	54	−34.5	−9	−5.03
	Temporal middle gyrus	Ipsilateral	285				
Cluster 3	*Parietal lobe*	Ipsilateral	291	19.5	−52.5	24	−4.21
	Precuneus	Ipsilateral	181				
Cluster 4	*Parietal lobe*	Contralateral	266	−43.5	−28.5	24	−3.97
	Rolandic operculum	Contralateral	114				
	Supramarginal	Contralateral	50				
	Postcentral	Contralateral	42				

### Correlation between regional brain atrophy and clinical motor impairment

We performed partial correlation analyses to detect relationships between regions showing brain atrophy and motor impairment scores (Figure 2B). Age, sex, brain flipping history (binary variable) and MMSE score were regressed out as covariates of no interest. Among those regions, we observed that lower deformation values in contralateral MTG were correlated with higher UPDRS III scores (*r* = −0.506, *P* = 0.007), motor scores from affected limbs (*r* = −0.393, *P* = 0.043) and axial motor scores (*r* = −0.561, *P* = 0.002). Due to the heterogeneity of motor impairment, we also detected a negative correlation between deformation values in this region and akinesia/rigidity (*r* = −0.503, *P* = 0.007) but not tremor (*r* = −0.017, *P* = 0.933).

### Altered structural network topology in PD patients with hemiparkinsonism

#### Small-world parameters and group comparisons

**Figure 3:**
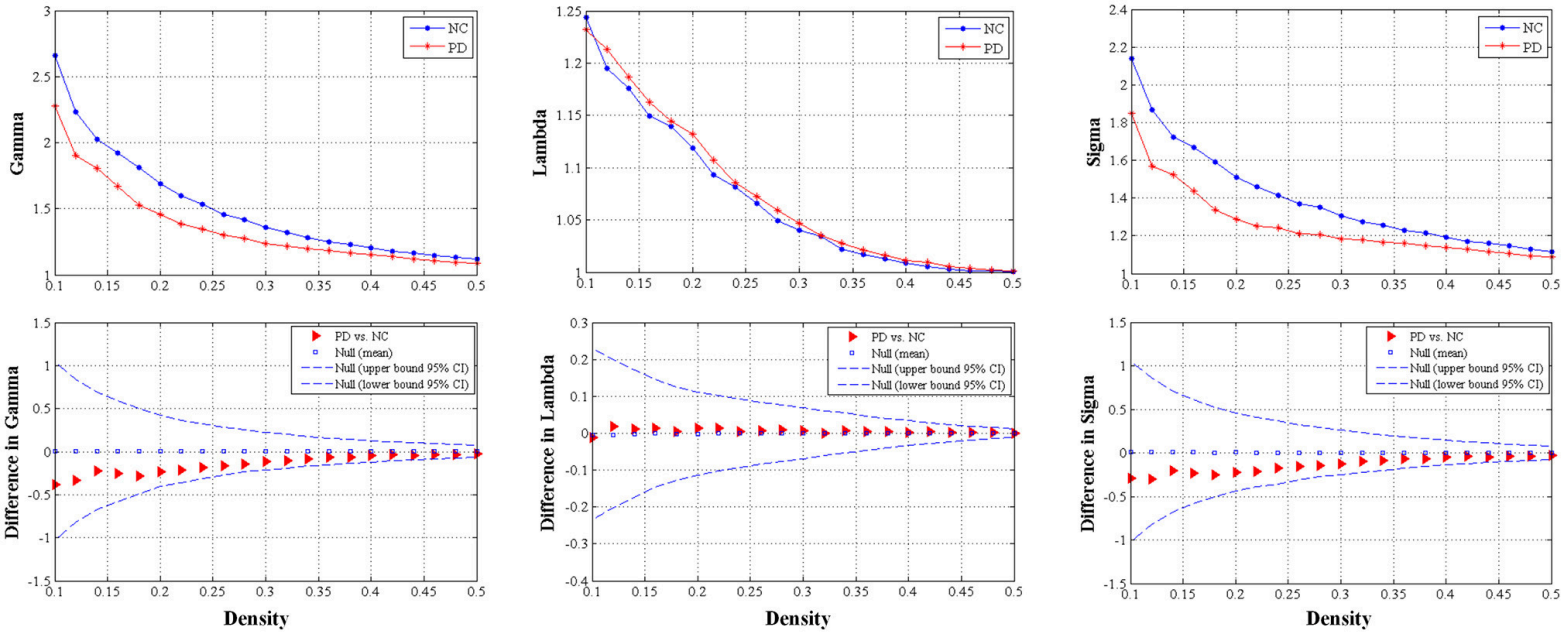
The distributions **(upper row)** and intergroup differences **(bottom row)** of small-worldness including Gamma, Lambda and Sigma in/between normal controls and PD patients with hemiparkinsonism. The upper and lower 95% confidence intervals were shown with dotted line in the bottom row. Thus, no significant difference was observed between groups.

We found that over the density range of 0.1–0.5, the Gamma were larger than 1 and the Lambda were nearly 1 (Sigma = Gamma/Lambda was consistently greater than 1) for both groups of structural networks (Figure 3). Therefore, the present study demonstrated that these SCNs from PD and control groups exhibited typical features of small-worldness and there was no significant intergroup difference in AUC analyses (*P* = 0.317 for Gamma, *P* = 0.841 for Lambda, and *P* = 0.403 for Sigma) (Figure 3).

#### Global network measures comparisons

**Figure 4:**
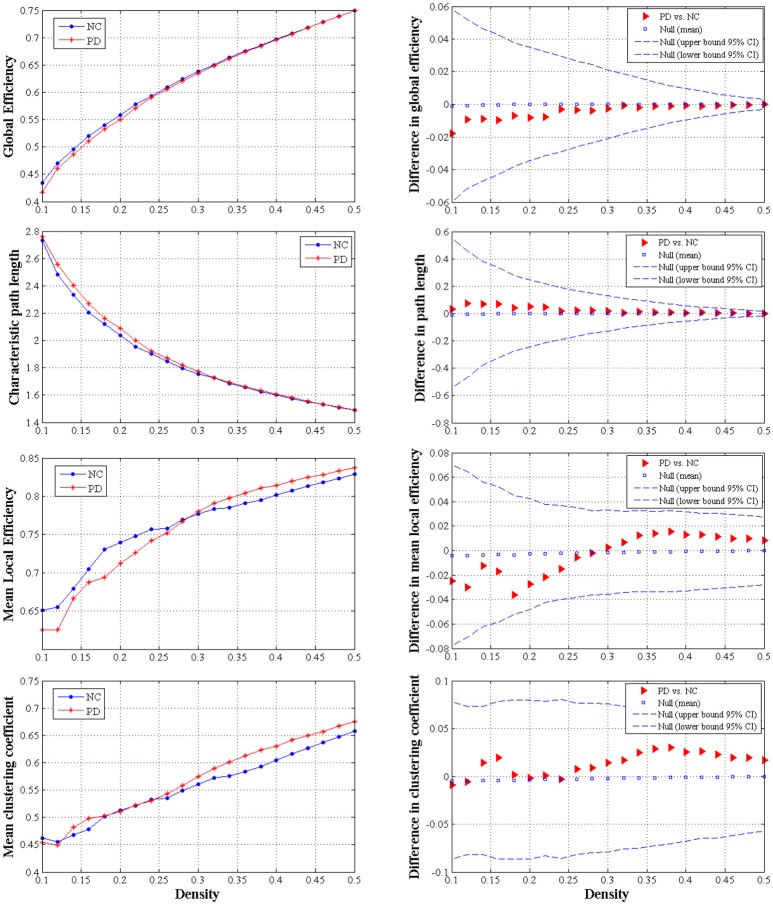
The distributions **(left column)** and intergroup differences **(right column)** of global measures including global efficiency, characteristic path length, mean local efficiency and mean clustering coefficient in/between normal controls and PD patients with hemiparkinsonism. The upper and lower 95% confidence intervals were shown with dotted line in the right column. No significant difference was observed between groups.

We did not observe any significant difference of global network measures through AUC analyses, e.g., global efficiency (*P* = 0.721), the characteristic path length (*P* = 0.730), local efficiency (*P* = 0.912) and clustering coefficient (*P* = 0.676), between PD patients and normal controls (Figure 4). This indicated that global measures of SCN were fairly preserved during the early hemiparkinsonism.

#### Regional network measures comparisons

**Figure 5:**
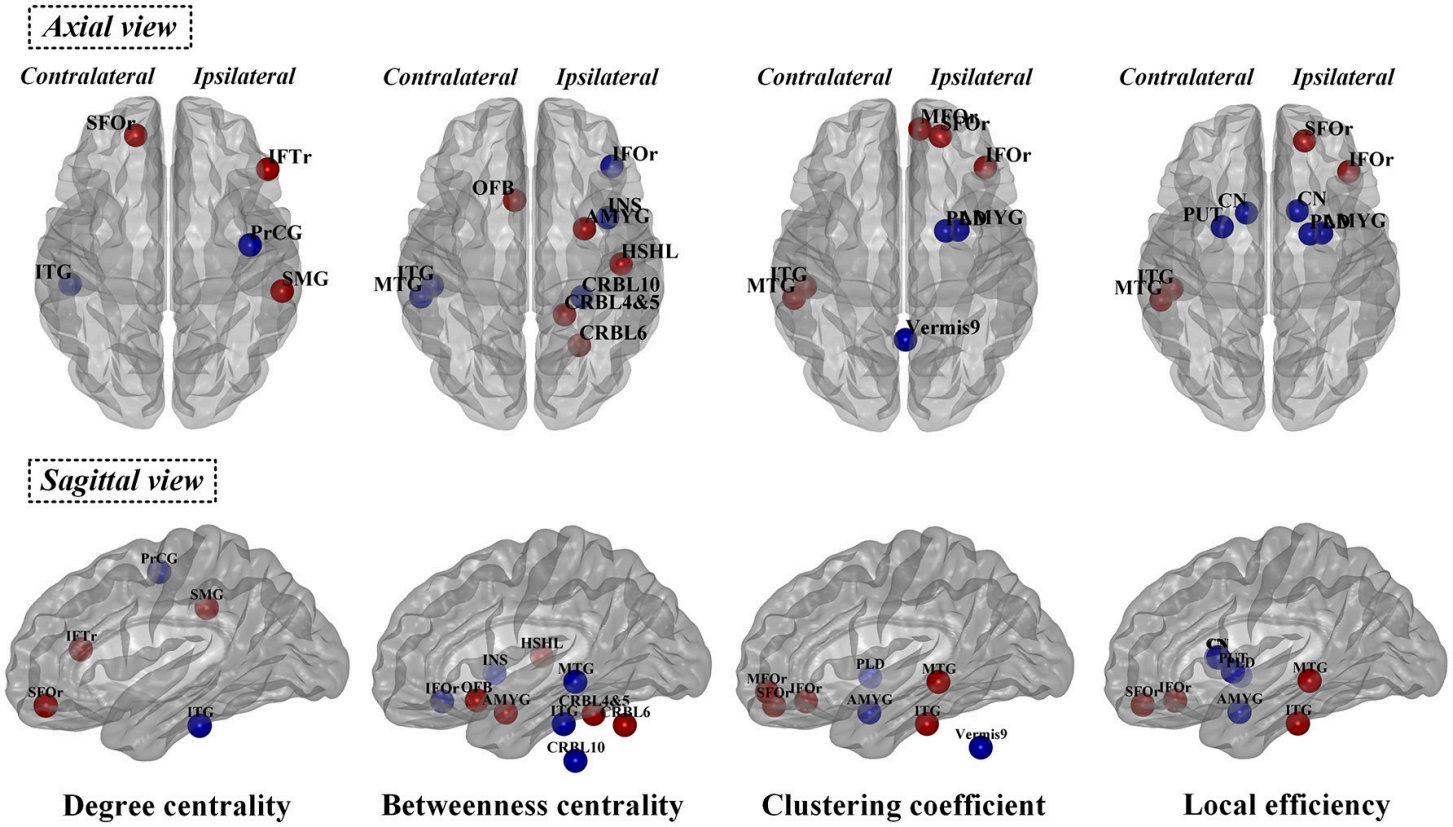
Significant differences of normalized nodal properties including betweenness centrality, degree centrality, clustering coefficient and local efficiency between PD patients with hemiparkinsonism and normal controls. Both axial view and sagittal view were shown. Red color showed the increased nodal values in patient group and blue color showed the decreased nodal values in patient group compared with normal controls. SFOr, superior orbitofrontal gyrus; IFTr, triangular part of inferior frontal gryus; MFOr, middle orbitofrontal gryus; SFOr, superior orbitofrontal gyrus; IFOr, inferior orbitofrontal gryus; OFB, olfactory gyrus; PrCG, precentral gyrus; MTG, middle temporal gyrus; ITG, inferior temporal gyrus; CN, caudate nucleus; PUT, putamen; PLD, pallidum; AMYG, amygdala; INS, insula; HSHL, Heschel's gyrus; SMG, supramarginal gryus; CRBL, cerebellum.

The observed between-group differences in regional network measures did not survive after correction for multiple comparisons. However, the uncorrected SCN results also revealed pathophysiological information of network function other than deformation analysis (Table 3 and Figure 5), thus we considered the following to be exploratory. Compared with normal controls, PD patients showed decreased nodal degree centrality in ipsilateral precentral gyrus and contralateral inferior temporal gyrus (ITG) but increased nodal degree centrality in ipsilateral interior frontal gyrus triangular part (IFTr) and supramarginal gyrus and contralateral superior orbitofrontal gyrus (SFOr). For nodal betweenness centrality, ipsilateral inferior orbitofrontal gyrus (IFOr), insula and cerebellum area 10 and contralateral MTG and ITG had lower values while ipsilateral amygdala, Heschel's gyrus, cerebellum area 4 and 5 and 6 and contralateral olfactory gyrus had higher values in PD than that in normal controls. In comparison with normal controls, ipsilateral amygdala and pallidum and vermis area 9 had decreased nodal clustering coefficient but ipsilateral IFOr, middle orbitofrontal gyrus (MFOr), SFOr and contralateral MTG and ITG had increased nodal clustering coefficient in PD patients. And, PD patients showed decreased nodal local efficiency in ipsilateral amygdala and pallidum, bilateral caudate and contralateral putamen but increased nodal local efficiency in ipsilateral SFOr and IFOr and contralateral MTG and ITG compared with normal controls. In summary, through analyzing nodal measures in SCN, we found that, (1) bilaterally nodal reorganization of SCN mainly composed of frontal, temporal, subcortex and cerebellum was explored in PD patients with hemiparkinsonism; (2) increased nodal properties could be commonly observed in frontal lobes, which was consistently seen in the hub analysis (shown in the below); (3) disruption of subcortex including basal ganglia and amygdala was detected by nodal local efficiency and clustering coefficient but not centralities, which indicated that the dysfunction in these regions was more likely localized in certain subgraph.

**Table 3:** Alterations of nodal measures between PD and normal controls.

**Node**	**Hemisphere**	**AUC value of Nodal property**			**PD vs. controls**
		**PD**	**Normal controls**	***P*-value**	
**DEGREE CENTRALITY**					
Precentral	Ipsilateral	0.192	0.611	0.039	–
Temporal_Inf	Contralateral	0.491	0.826	0.036	–
Frontal_Sup_Orb	Contralateral	0.795	0.409	0.030	+
Frontal_Inf_Tri	Ipsilateral	0.657	0.266	0.029	+
SupraMarginal	Ipsilateral	0.689	0.232	0.020	+
**BETWEENNESS CENTRALITY**					
Frontal_Inf_Orb	Ipsilateral	0.129	1.295	0.001	–
Insula	Ipsilateral	0.274	1.193	0.007	–
Temporal_Mid	Contralateral	0.258	0.806	0.050	–
Temporal_Inf	Contralateral	0.333	1.358	<0.001	–
Cerebellum_10	Ipsilateral	0.042	0.606	0.039	–
Olfactory	Contralateral	1.339	0.320	0.017	+
Amygdala	Ipsilateral	1.483	0.263	0.016	+
Heschel	Ipsilateral	0.922	0.345	0.018	+
Cerebellum_4_5	Ipsilateral	1.058	0.109	0.019	+
Cerebellum_6	Ipsilateral	1.643	0.664	0.039	+
**CLUSTERING COEFFICIENT**					
Amygdala	Ipsilateral	0.313	0.453	0.004	–
Pallidum	Ipsilateral	0.285	0.439	0.044	–
Vermis_9	/	0.338	0.596	0.010	–
Frontal_Inf_Orb	Ipsilateral	0.498	0.340	0.004	+
Frontal_Mid_Orb	Ipsilateral	0.484	0.381	0.044	+
Frontal_Sup_Orb	Ipsilateral	0.487	0.375	0.030	+
Temporal_Mid	Contralateral	0.465	0.361	0.026	+
Temporal_Inf	Contralateral	0.443	0.336	0.007	+
**NODAL LOCAL EFFICIENCY**					
Amygdala	Ipsilateral	0.377	0.426	0.047	–
Caudate	Contralateral	0.258	0.386	0.050	–
Caudate	Ipsilateral	0.261	0.391	0.038	–
Putamen	Contralateral	0.327	0.410	0.032	–
Pallidum	Ipsilateral	0.331	0.418	0.028	–
Frontal_Sup_Orb	Ipsilateral	0.447	0.395	0.031	+
Frontal_Inf_Orb	Ipsilateral	0.450	0.383	0.015	+
Temporal_Mid	Contralateral	0.438	0.394	0.033	+
Temporal_Inf	Contralateral	0.427	0.384	0.024	+

*/, Not available; –, PD patients < normal controls; +, PD patients > normal controls*.

#### Hub distribution

**Figure 6:**
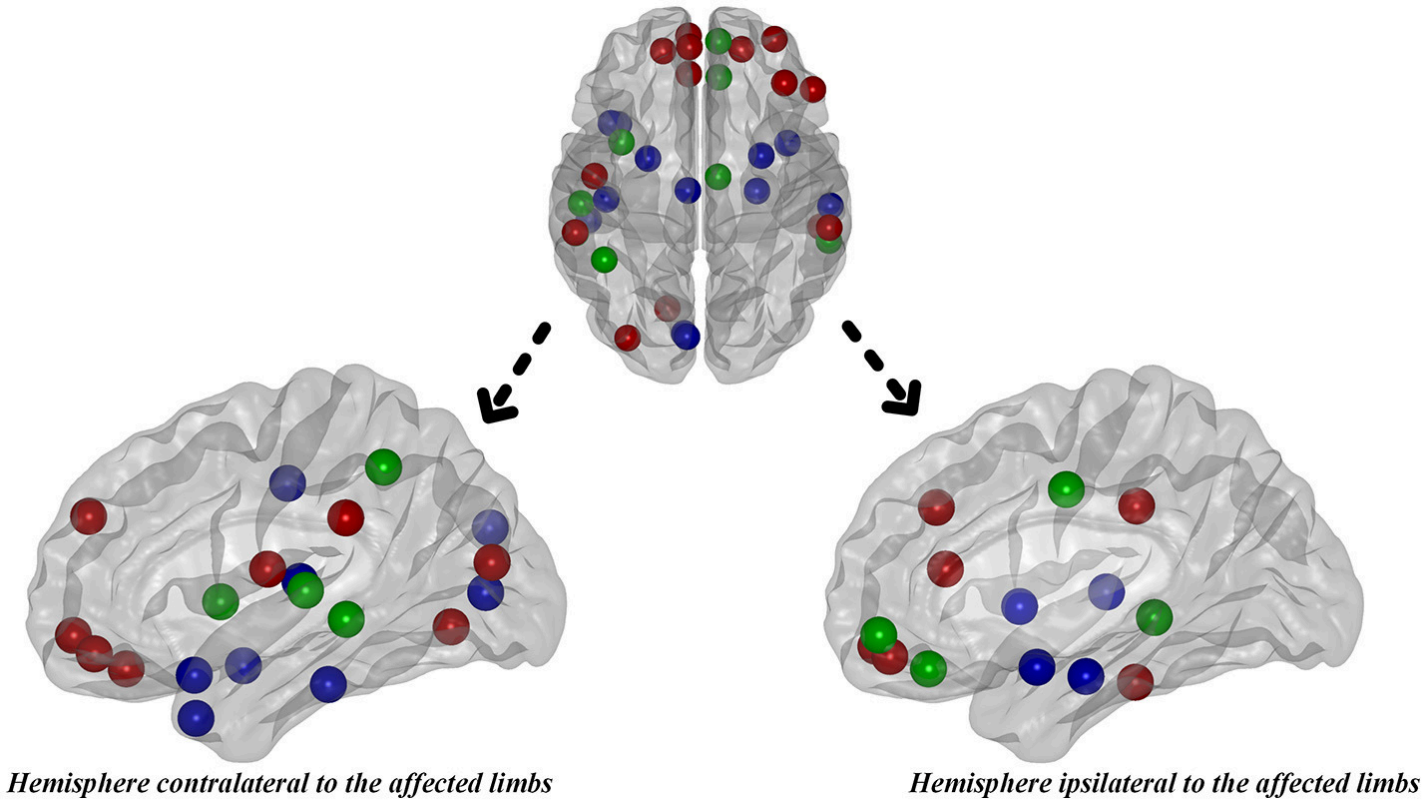
The distribution of hubs in PD patients with hemiparkinsonism. Green color represented the overlapped hubs in both groups; red color represented the regenerated hubs in patient group and blue color represented the disrupted hubs in patient group.

Hub analysis is a descriptive analysis since its statistical significance is unknown. Here, the hubs were identified as the nodes with degree centrality that was one SD above the network averages. As a result, 22 hubs in PD patients and 20 hubs in normal controls were detected in total. Eight hubs, all of which were assigned to paralimbic and heteromodal nodes, were overlapped in the two groups (Table 4 and Figure 6). Considering the hub distribution in normal controls as a reference, 12 hubs mainly assigned to paralimbic-limbic and heteromodal networks were disrupted and, alternatively, 14 hubs including eight frontal nodes were additionally detected in PD patients. Thus, reorganization of hub distribution could be observed in PD patients with hemiparkinsonism.

**Table 4:** The hub distributions of PD patients and normal controls.

	**Hub**	**Ipsi/Cont**	**Functional class**
**PD patients (22 hubs)**	**Overlapped Hubs In PD Patients and Normal Controls (8 Hubs)**		
	Cingulum_Mid	Ipsi	Paralimbic
	Frontal_Med_Orb	Ipsi	Paralimbic
	Insula	Cont	Paralimbic
	Parietal_Inf	Cont	Heteromodal (Association)
	Rectus	Ipsi	Paralimbic
	Temporal_Mid	Cont	Heteromodal (Association)
	Temporal_Mid	Ipsi	Heteromodal (Association)
	Temporal_Sup	Cont	Heteromodal (Association)
	**Regenerated Hubs in PD Patients (14 Hubs)**		
	Frontal_Inf_Tri	Ipsi	Heteromodal (Association)
	Frontal_Med_Orb	Cont	Paralimbic
	Frontal_Mid_Orb	Ipsi	Paralimbic
	Frontal_Mid	Ipsi	Heteromodal (Association)
	Frontal_Sup_Medial	Cont	Heteromodal (Association)
	Frontal_Sup_Orb	Cont	Paralimbic
	Frontal_Sup_Orb	Ipsi	Paralimbic
	Lingual	Cont	Unimodal (Association)
	Occipital_Mid	Cont	Unimodal (Association)
	Rectus	Cont	Paralimbic
	Rolandic_Oper	Cont	Association
	SupraMarginal	Cont	Heteromodal (Association)
	SupraMarginal	Ipsi	Heteromodal (Association)
	Temporal_Inf	Ipsi	Heteromodal (Association)
**Normal controls (20 hubs)**	**Overlapped Hubs in PD Patients and Normal Controls (8 Hubs)**		
	Cingulum_Mid	Ipsi	Paralimbic
	Frontal_Med_Orb	Ipsi	Paralimbic
	Insula	Cont	Paralimbic
	Parietal_Inf	Cont	Heteromodal (Association)
	Rectus	Ipsi	Paralimbic
	Temporal_Mid	Cont	Heteromodal (Association)
	Temporal_Mid	Ipsi	Heteromodal (Association)
	Temporal_Sup	Cont	Heteromodal (Association)
	**Disrupted Hubs in PD Patients (12 Hubs)**		
	Amygdala	Cont	Limbic
	Amygdala	Ipsi	Limbic
	Calcarine	Cont	Primary
	Cingulum_Mid	Cont	Paralimbic
	Cuneus	Cont	Heteromodal (Association)
	Heschel	Cont	Primary
	Insula	Ipsi	Paralimbic
	Parahippocampal	Ipsi	Paralimbic
	Temporal_Inf	Cont	Heteromodal (Association)
	Temporal_Pole_Mid	Cont	Paralimbic
	Temporal_Pole_Sup	Cont	Paralimbic
	Temporal_Sup	Ipsi	Heteromodal (Association)

*The hubs were identified as the nodes with degree centrality that was one SD above the network averages. As a result, in PD patients with hemiparkinsonism, 22 hubs were observed in total including eight overlapped hubs and 14 regenerated hubs compared with normal controls. Among the 20 hubs observed in normal controls, 12 hubs disrupted in PD patients. Ipsi, ipsilateral hemisphere to the affected limbs; Cont, contralateral hemisphere to the affected limbs*.

## Discussion

Hemiparkinsonism duration of PD patients is a key time window to study early pathology. Though only structural data was used in the present study, we could potentially make a comprehensive exploration to understand the pathomechanism of PD patients with hemiparkinsonism anatomically and functionally. We mainly presented two findings: first, brain atrophy in bilateral temporoparietal regions was detected in the PD patients at the early hemiparkinsonism phase, and the lower deformation values in contralateral MTG were correlated with the higher motor impairment scores which was dominated by akinesia/rigidity; second, bilaterally nodal reorganization of SCN was widely detected, e.g., enhanced frontal function but disrupted basal ganglia function, while global properties and small-worldness were preserved in PD patients with hemiparkinsonism.

### Brain atrophy observed by DBM in PD patients with hemiparkinsonism

The deformations value facilitates a quantitative description of local variability in subjects along with a multivariate statistical analysis (Borghammer et al., 2010). Here, we observed atrophy of bilateral MTG, ipsilateral precuneus and contralateral Rolandic operculum extending to supramarginal gyrus and postcentral gyrus in nondemented PD patients with hemiparkinsonism. Nevertheless, the exploratory analyses failed to remain statistically significant following conservative corrections for multiple comparisons; power may be limited by sample size and the modest degree of atrophy seen in early stages of hemiparkinsonism (Fereshtehnejad et al., 2017). To the best of our knowledge, this was the first time that DBM measures of atrophy were found to detect pathomechanism of hemiparkinsonism. Previously, Borghammer et al., conducted the first DBM study in a cohort of early PD patients and merely found cerebellum atrophy (Borghammer et al., 2010). Recently, Zeighami et al. reported atrophy of basal ganglia network through decomposing DBM data using independent component analysis (Zeighami et al., 2015). The same team also found a progressive pattern of brain atrophy with the disease exacerbation by performing DBM without multiple comparison correction (Fereshtehnejad et al., 2017). This study detected atrophy in temporal and parietal lobes which was partially consistent with our finding. We speculated that the existing discrepancies might be resulting from the different stages of PD population, e.g., recruiting patients with bilateral symptoms (Hoehn and Yahr Stage = 2; Borghammer et al., 2010; Fereshtehnejad et al., 2017), different processing and statistical methods and sample sizes (Zeighami et al., 2015), and the specific designment for hemiparkinsonism in the present study.

To date, the only existing MRI study on PD patients with hemiparkinsonism reported no GM abnormality by volume-based morphometry with conservative corrections for multiple comparisons (Agosta et al., 2014). Conversely, consistent with our study, it has been well established that neocortex atrophy occurs in MTG, precuneus, Rolandic operculum, supramarginal gyrus and postcentral gyrus in mild to moderate PD patients (Lyoo et al., 2010; Pereira et al., 2012; Agosta et al., 2013; Zarei et al., 2013), though several explorative studies did not report any structural alterations in PD patients even with a higher UPDRS III score (25.8 ± 11.1; Burton et al., 2004, and 20.3 ± 8.9; Weintraub et al., 2011) than us (14.2 ± 5.7). That might demonstrate the sensitive measurement of brain structure by DBM. Pathologically, the distribution of brain atrophy observed in the present study was overlapped with the area where cortical Lewy bodies and Lewy neurites were found in patients with symptomatic PD patients, corresponding to Braak's stage 4 and 5 (Braak et al., 2003; Braak and Del, 2008). Moreover, *in vivo* metabolic studies confirmed the hypometabolism in temporoparietal regions (Eberling et al., 1994; Hu et al., 2000). Intriguingly, we observed that, more severe atrophy in contralateral MTG implied worse motor impairment. Akinesia/rigidity but not tremor had this specific correlation, which suggested that different pathogeneses might be underlying these different motor impairments as previous studies reported (Zaidel et al., 2009; Guan et al., 2017a,b).

Taken together, mild brain atrophy restricted to bilateral temporoparietal regions could be investigated by DBM in the early parkinsonism period. Deformation information of MTG contralateral to the affected limbs might be a potential living biomarker to monitor disease progression.

### Reorganization of structural network in PD patients with hemiparkinsonism

SCN could provide potentially useful functional information other than DBM. Human brain is a complex and interconnected system with important topological attributes, such as small-worldness, high regional and global efficiency and highly connected hubs (Watts and Strogatz, 1998; Liao et al., 2017), and PD tends to be recognized as a neurodegenerative disease with multi-layer disconnection (Cerasa et al., 2016). As global measures, clustering coefficient representing segregation (the ability for specialized processing to occur within densely interconnected groups of brain regions) and characteristic path length representing brain integration (the ability to rapidly combine specialized information from distributed brain regions) are important properties in human brain (Rubinov and Sporns, 2010; Liao et al., 2017). In the present study, we did not find any significant change in above measures as well as small-worldness in patients with hemiparkinsonism compared with normal controls. In accordance with present study, no significant difference of global properties and small-worldness was observed in early PD patients while decreased global efficiency was specifically observed in PD patients with mild cognitive impairment (Pereira et al., 2015). Therefore, at clinically early stage without cognitive impairment, brains of PD patients with hemiparkinsonism still keep a relatively integrated ability for information processing within and across anatomically interconnected brain regions.

Nodal properties are mainly assigned to two different measures like nodal centralities from globally densely connected network (i.e., nodal degree centrality and betweenness centrality) and nodal properties from localized subgraph information (i.e., nodal clustering coefficient and local efficiency) according to their definitions (Freeman, 1978; Latora and Marchiori, 2001; Rubinov and Sporns, 2010; Liao et al., 2017). Though the previous study did not find any nodal alterations in early PD patients without cognitive impairment (Pereira et al., 2015), we observed bilateral enhanced nodal measures in multiple frontal nodes of PD patients with hemiparkinsonism, which could be probably supported by the enhanced frontal function found in PD patients (Cools et al., 2010). Further, dysfunction of basal ganglia network (subgraph) could be regarded as a hallmark of PD (Obeso et al., 2000; Szewczyk-Krolikowski et al., 2014; Guan et al., 2017b) while enhanced nodal centrality was oppositely observed in basal ganglia nodes (Guan et al., 2017b; Ko et al., 2017). Consistent with our SCN results, we observed significant reduction of nodal properties specifically belonging to the subgraph properties (nodal clustering coefficient and local efficiency) in caudate, putamen and pallidum of PD patients with hemiparkinsonism. Thus, SCN analysis has the ability to seize PD core pathogenesis. Interestingly, we also observed a shift between nodal centralities and localized properties, for example, decreased centralities but increased nodal clustering coefficient and local efficiency in contralateral ITG and MTG, which disclosed a possible explanation that in order to rebalance the disrupted nodal centralities these nodes were getting closer in local subgraphs. Though cerebellum function is not fully known, evidence confirmed the pathological changes in cerebellum caused by the degeneration of nigrostriatal dopaminergic neurons (Rolland et al., 2007; Borghammer et al., 2010) and its complementary role in modulating cerebral dysfunction (Wu and Hallett, 2013; Guan et al., 2017b; Zeng et al., 2017). In the present study, the coexistence of enhanced and disrupted nodes in cerebellum again provided new evidence to support the pathological and complementary alterations in PD patients with hemiparkinsonism.

Hubs in the brain networks are regions with multimodal or integrative function and their disturbances can remarkably influence the stability and efficiency of the network (van den Heuvel and Sporns, 2013). A majority of hubs observed in temporal cortex, frontal cortex, cingulate cortex, parietal cortex and occipital cortex were largely in accordance with the previous literature (van den Heuvel and Sporns, 2013; Pereira et al., 2015). We observed that, 12 hubs, most of which were assigned to limbic-paralimbic and association nodes, disrupted in PD patients compared with normal controls. As pathologically studied, limbic-paralimbic and association cortex were gradually involved by α-synuclein-immunopositive Lewy neurites and Lewy bodies following an upward progressive order (Braak et al., 2003; Braak and Del, 2008), which hinted that even if hemiparkinsonism duration is clinically early, the pathological progression could be at an advanced stage. Alternatively, 14 regenerated hubs, mainly composed of frontal nodes, were observed in PD patients, which was consistent with studies explaining that the increase of frontal hubs might stem from upregulated frontal dopaminergic transmission in early PD in response to reductions in striatal dopamine (Cools et al., 2010; Pereira et al., 2015). A recent graph study using metabolic data also highlighted the high centrality derivatives in a majority of frontal nodes (Ko et al., 2017). In brief, we supposed that the bilateral remapping of hub distribution in PD patients with hemiparkinsonism came from the jointing effect of the pathological infiltration of Lewy neurites and Lewy bodies and functionally complementary alterations on rebalancing the cerebral dysfunction.

Several limitations in the present study should be noted. First, for the restriction of sample size of unilateral onset patients, we had flipped the raw images from left-onset patients before imaging preprocessing. Therefore, the influence resulting from original inter-hemispherical differences might be inevitable. While the previous study indicated different structural substrates between left-onset and right-onset patients (Lee et al., 2015), only relatively homogeneous data after flipping unaffected hemisphere into affected side could we observe the disease-related alterations and minimize the underlying difference leading from complex brain reorganization. Also, it has been noted that durative uptake of anti-parkinsonian medicine would, to some extent, reorganize brain structure and function (23 patients in the present study). Further studies with drug-naïve patients could help solve this issue. Finally, axial motor impairment should be related to brain changes, which might be a potential factor influencing the supposed “unaffected hemisphere.” As a result, only deformation values in contralateral MTG were showing significantly negative correlation with that.

In conclusion, mild brain atrophy in the temporoparietal regions and widespread reorganization of structural network, e.g., enhanced frontal function and disruption of basal ganglia nodes, occurred in both hemispheres in PD patients with hemiparkinsonism. Also, the MTG contralateral to the affected limbs expressing clinically correlated brain atrophy might be a potential living biomarker to monitor disease progression. The fact that results from DBM did not completely coincide with the significant regions of our network analysis was probably related with structural networks containing exclusive information that could not be captured by traditional structural analyses. Therefore, the combination of DBM and SCN analyses can provide a comprehensive and sensitive evaluation for early PD patients with hemiparkinsonism.

## Author contributions

All of the coauthors listed meet the criteria for authorship. XX and XG were involved with study concept and design, acquisition of data, analysis and interpretation of data, drafting/revising the manuscript. TG, QZ, RY, JW, JZ, MX, QG, and PH were involved with acquisition of data, analysis and interpretation of data. JP and BZ were involved with PD patients' recruitment. MZ was involved with drafting/revising the manuscript. XX and MZ were responsible for obtaining funding and supervision of study.

### Conflict of interest statement

The authors declare that the research was conducted in the absence of any commercial or financial relationships that could be construed as a potential conflict of interest.
